# Minimally Invasive Biological Gracilis Augmentation of Fascia Lata for Intra‐ and Extra‐articular Anterior Cruciate Ligament Reconstruction: XO FAST Technique

**DOI:** 10.1002/atn2.70303

**Published:** 2026-11-12

**Authors:** Pierre Denis‐Aubrée, Serge Herman, Alexandre Hardy, Yoann Bohu

**Affiliations:** ^1^ Clinique du Sport Paris France; ^2^ Société Française de Chirurgie du Sport Paris France

## Abstract

The fascia lata (FL) is a long‐established and proven graft for anterior cruciate ligament reconstruction, allowing intra‐ and extra‐articular reconstruction to be combined with a single graft and a single femoral tunnel, without femoral fixation. Despite its biomechanical and clinical advantages, its use has been limited by the morbidity of the donor site and the size of the incision. We describe a minimally invasive technique for anterior cruciate ligament reconstruction using a pedicled FL strip reinforced with the gracilis tendon, which reduces donor‐site morbidity while maintaining the benefits of combined reconstruction. This technique is based on reproducible anatomical landmarks for harvesting the FL, a limited lateral, and simplified graft preparation. The graft allows optimal control of anterior and rotational stability of the knee while preserving the extensor apparatus and the semitendinosus tendon. The absence of femoral fixation, the use of a single femoral tunnel, and a single tibial interference screw make this technique particularly suitable for pediatric surgery, revision surgery, and situations with a high risk of rerupture. This technical note presents a reliable and reproducible alternative to conventional techniques for reconstructing the anterior cruciate ligament using the FL, limiting morbidity while optimizing stability and functional outcomes.

## Graphical abstract

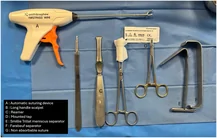

**VIDEO 1 atn270303-fig-1001:** Right knee, supine position. First stage of the operation: the gracilis muscle is harvested via a vertical skin incision opposite the anterior tibial tuberosity after opening the sartorius muscle longitudinally using an open stripper, removing the muscle body, folding it to obtain a single bundle measuring 10 to 12 cm in length and 4 to 5 mm in diameter, and impregnating it with vancomycin. Second stage of the operation: the fascia lata (FL) is harvested via a lateral approach with a longitudinal incision along the skin landmarks between the lateral epicondyle and the head of the fibula; then, a 15 to 20 mm wide strip of FL is harvested, leaving it pedicled distally. Third stage of the operation: the entry point of the femoral tunnel is marked from the outside in by a square‐tipped predrilled hole located behind the lateral collateral ligament and posterior to the Lemaire vessels, anterior to the lateral femorotibial space. The entry point of the tunnel is marked on the FL to secure the gracilis from the femoral tunnel and prevent the graft from becoming tubular outside the femoral tunnel. Fourth stage of the operation: preparation of the graft, the FL is augmented by the gracilis, fixed with a nonabsorbable suture, and then calibrated to a diameter usually between 7.5 and 8.5 mm. Video content can be viewed at https://doi.org/10.1002/atn2.70303.

The fascia lata (FL) is a graft of choice for anterior cruciate ligament (ACL) reconstruction. In 1917, Hey Grove, then Alwyn Smith, used a FL graft for ACL reconstruction.^1^ The technique was then modified several times by MacIntosh, then Jaeger, to optimize the position of the femoral tunnel and facilitate closing of the FL.^2^, ^3^, ^4^, ^5^, ^6^ Later, Khiami described harvesting the FL through 2 lateral incisions to limit the size of the incision.^7^ Finally, more recently, Dos Santos et al.^8^ reported augmenting the intra‐articular portion of the iliotibial band by suturing the gracilis tendon (G). The main advantage of this technique is that intra‐articular and extra‐articular ACL reconstruction can be obtained with a single graft, a single femoral tunnel, and no femoral fixation.[Fig atn270303-fig-1001]

It has been confirmed that the addition of an extra‐articular graft decreases the rate of graft retears and secondary meniscectomies.^9^ Although the FL is a commonly used graft for ACL reconstruction,^10^ the technical difficulty of harvesting, graft preparation, the size of the incision, and the length of the procedure have limited its use.

XO FAST provides reproducible anatomical landmarks for the incision and for harvesting, as well as an incision that is comparable to that with the FL tenodesis technique.

## SURGICAL TECHNIQUE

### Material

This surgical technique requires the same surgical instruments as those for ACL reconstruction, as well as several other specific instruments (Figure 1).

**FIGURE 1:**
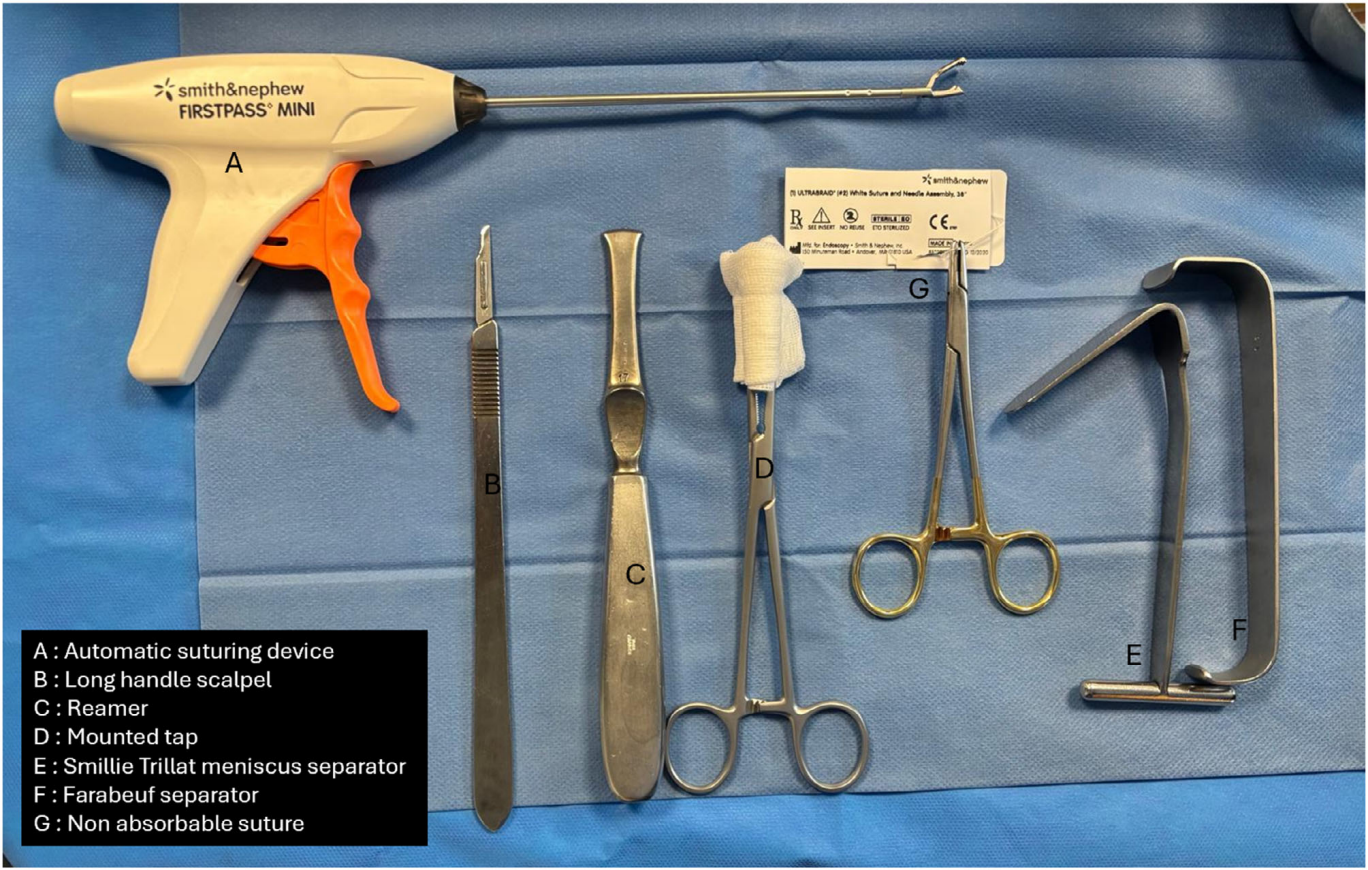
Material needed for this technique. (A) Automatic suturing device. (B) Long‐handle scalpel. (C) Reamer. (D) Mounted tap. (E) Smillie‐Trillat meniscus separator. (F) Farabeuf separator. (G) Nonabsorbable suture.

### Installation

The patient is positioned supine with the knee flexed to 75°. The anatomical skin landmarks include the lateral epicondyle (ECL), Gerdy's tubercle (Ge), pes anserinus, and the head of the fibula (F) (Figure 2, Video 1). The distance between the ECL and the F (b) is measured. This makes it possible to adapt the length of the harvested FL to the size of the patient (Figure 2). The length of the harvested FL strip should be twice the distance b plus the distance between the F and Ge (Figure 2).

**FIGURE 2:**
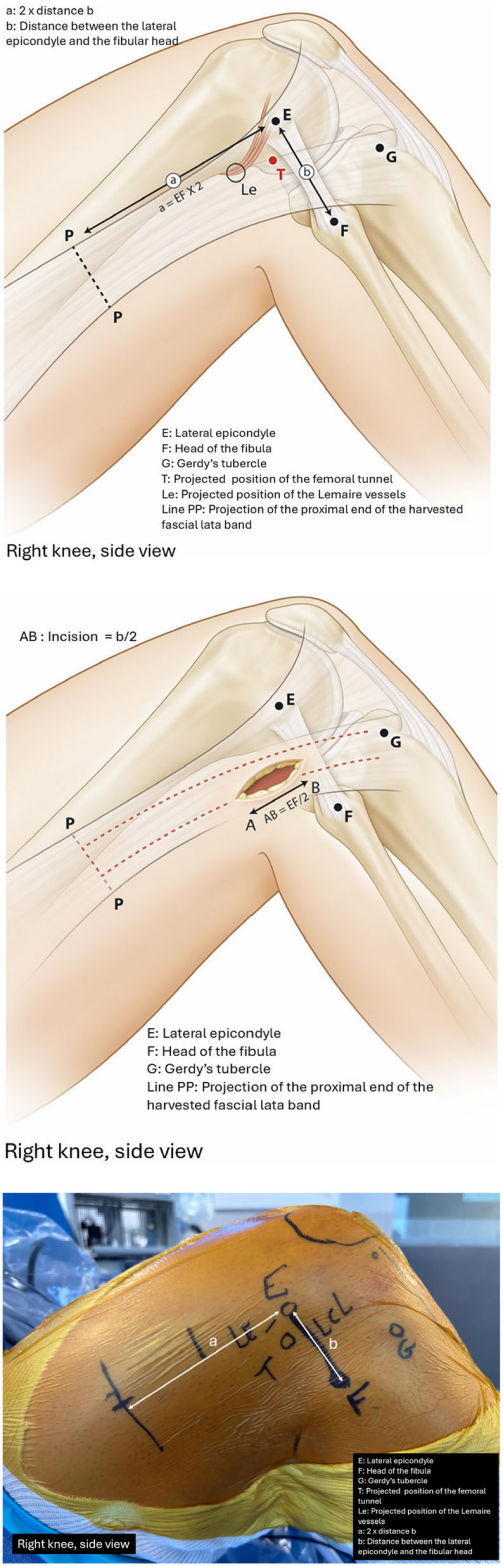
Right knee, supine position. Anatomical skin landmarks. E: Lateral epicondyle. F: Head of the fibula. G: Gerdy's tubercle. T: Projected position of the femoral tunnel. Le: Projected position of the Lemaire vessels. a: 2 × distance b. b: Distance between the lateral epicondyle and the fibular head. AB: Incision = b/2. Line PP: Projection of the proximal end of the harvested fascia lata band.

### Harvesting the Graft

First, the gracilis is harvested by making a 2‐cm vertical tibial incision centered on the pes anserinus. The aponeurosis of the sartorius muscle is exposed, and the G is isolated with a dissector and then harvested using a tendon stripper. The tendon is freed from its tibial insertion, the muscle fibers are removed and then it is folded into 2 strands around a suture, and the ends are threaded with a second braided absorbable suture (Ethicon, Raritan).

The FL is harvested after a longitudinal incision is made on the lateral side of the thigh and using the following landmarks: the distal end is the axial line ECL and F. The proximal end is located in the middle of the distance between ECL and F (distance b) (Figure 2). After an incision is made, the fascia superficialis is isolated superficially to expose the FL and dissected using a rugine from Ge to the previously identified landmark for the proximal end of the harvesting (Figure 2). The FL is exposed during the incision using 2 specific surgical separators: 2 small Farabeuf separators, distally, and a Smillie‐Trillat meniscus separator and a large Farabeuf separator, proximally. A longitudinal 15 to 20 cm wide strip of the FL is harvested starting from Ge with a #11 long‐handle scalpel. The length of the harvested FL strip should be twice the distance b plus the distance between the F and Ge (Figure 3). The posterior incision is made to leave 8 mm of FL for closing. The incision is first posterior, distally to proximally, then anterior to the condyle, where the position and type of separators are changed to expose the distal portion. The FL graft is dissected with Metzenbaum scissors around the end of the distal insertion of the lateral vastus, near the fat pads across from 3 parallel vessels called the Lemaire vessels, then freed proximally to distally, where it is left pedicled to the Ge (Figure 3).

**FIGURE 3:**
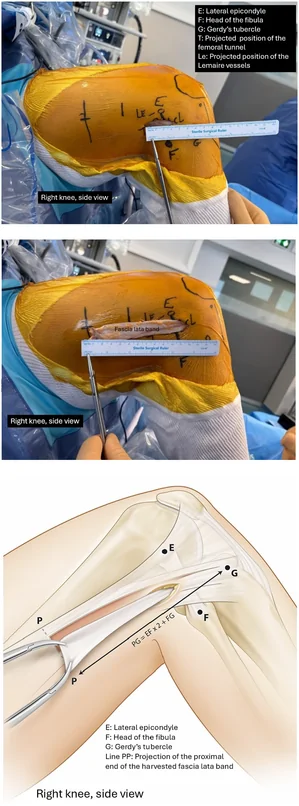
Right knee, supine position. Harvesting the fascia lata band.

### Identifying the Entry Point of the Femoral Tunnel

The entry point for outside‐in drilling of the femoral tunnel is identified. The anatomical landmarks are the lateral collateral ligament, the joint line, and the lateral femoral epicondyle. A square point is used to create a landmark above the joint line, behind the lateral collateral ligament, and posterior to the abovementioned Lemaire vessels (Figure 4).

**FIGURE 4:**
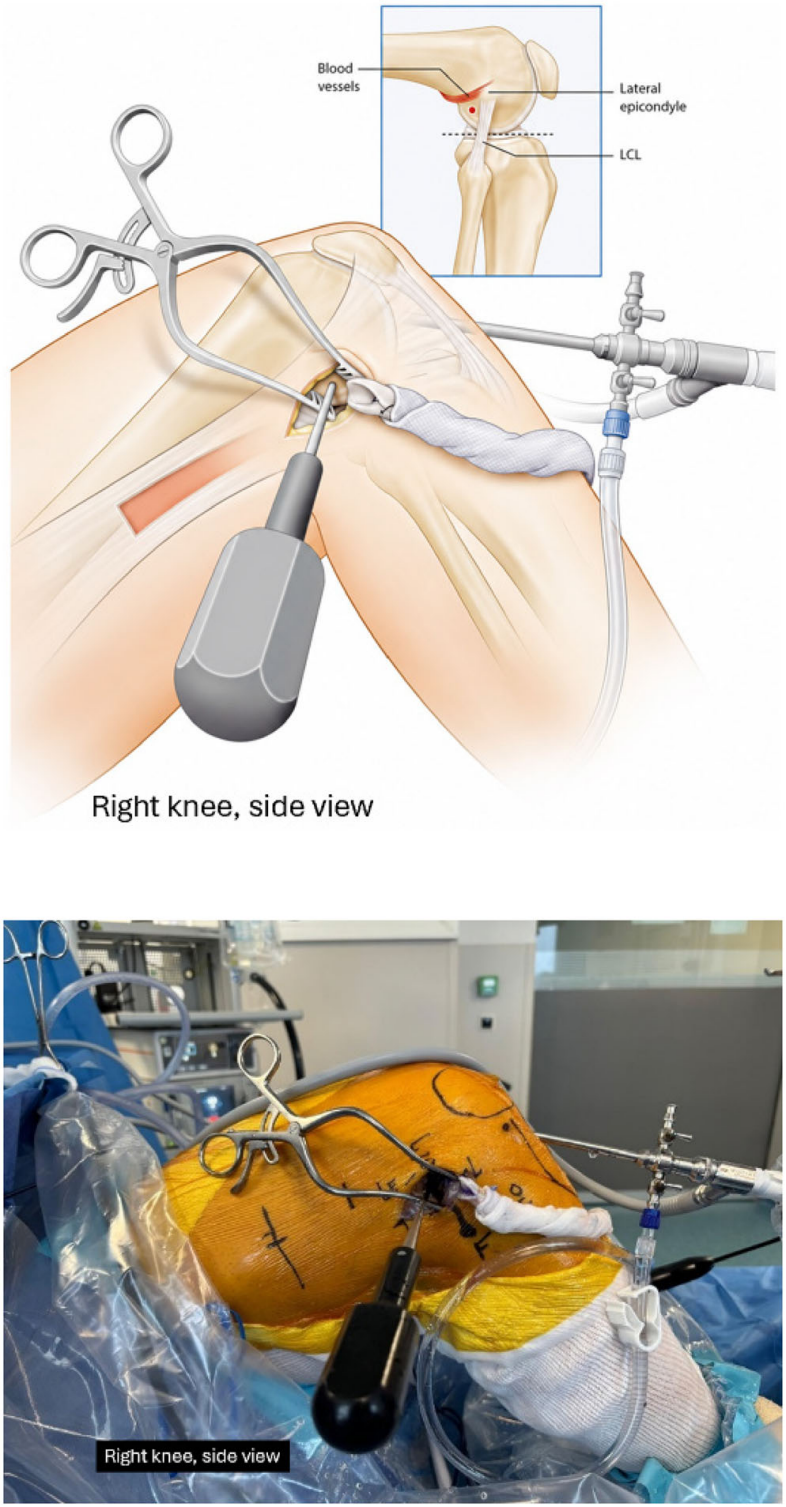
Right knee, supine position. Identifying the isometric point landmark. (LCL, lateral collateral ligament.)

### Preparing the Graft

The FL graft is augmented with 2 folded strands of the G, tubularized into an oval shape using braided absorbable sutures (Ethicon), and then sutured onto nonabsorbable traction sutures, UltraBraid (Smith & Nephew). The portion of the FL graft that is outside the femoral tunnel is not reinforced with the G and is flat, not tubular. The point where the FL enters the tunnel should be indicated with a dermatological landmark. The length and diameter of the graft are measured to determine the size of the bone tunnels (size for size) and interference screws (2 mm more than the measured size) (Figure 5).

**FIGURE 5:**
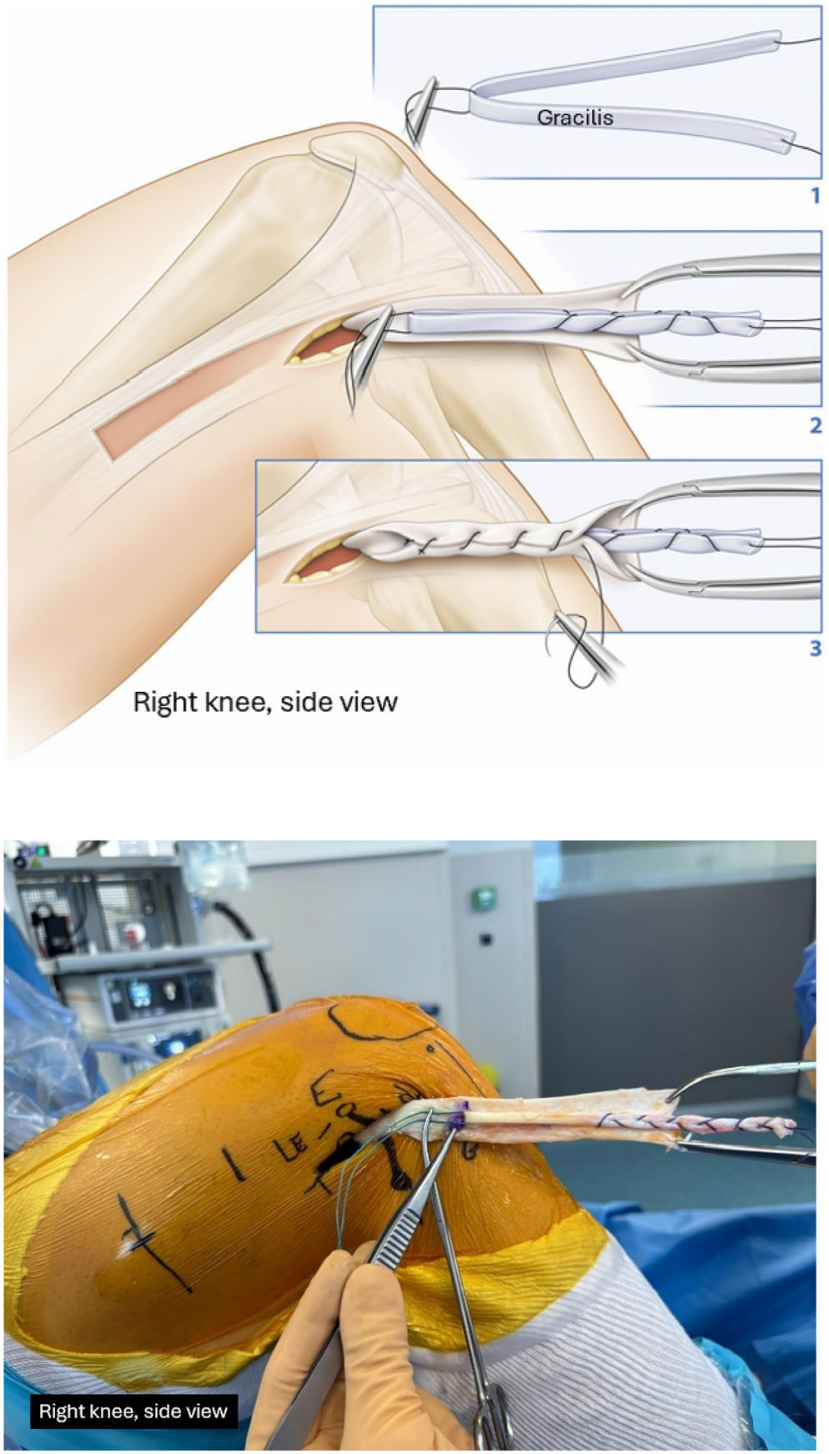
Right knee, supine position. Graft preparation.

### Arthroscopy

The arthroscopic part of the procedure begins with exploration of the joints and then creation of the tunnels.

### Creating the Femoral Tunnel

The outside‐in femoral tunnel is created using a drill guide inserted into the anterolateral portal. The exit point of the guide pin of the femoral tunnel is located 7 mm cortex of the femoral condyle. The exit point of **the guide pin of the femoral tunnel** is located very close to the posterior cortex of the femoral condyle. During flexion, it is located at 8 and 4 o'clock on the posterior edge of the intercondylar notch of the right and left knees, respectively. The guide pin is positioned, and the femoral tunnel is drilled using a 5‐mm drill bit first, then a second the same size as the graft (Figure 6).

**FIGURE 6:**
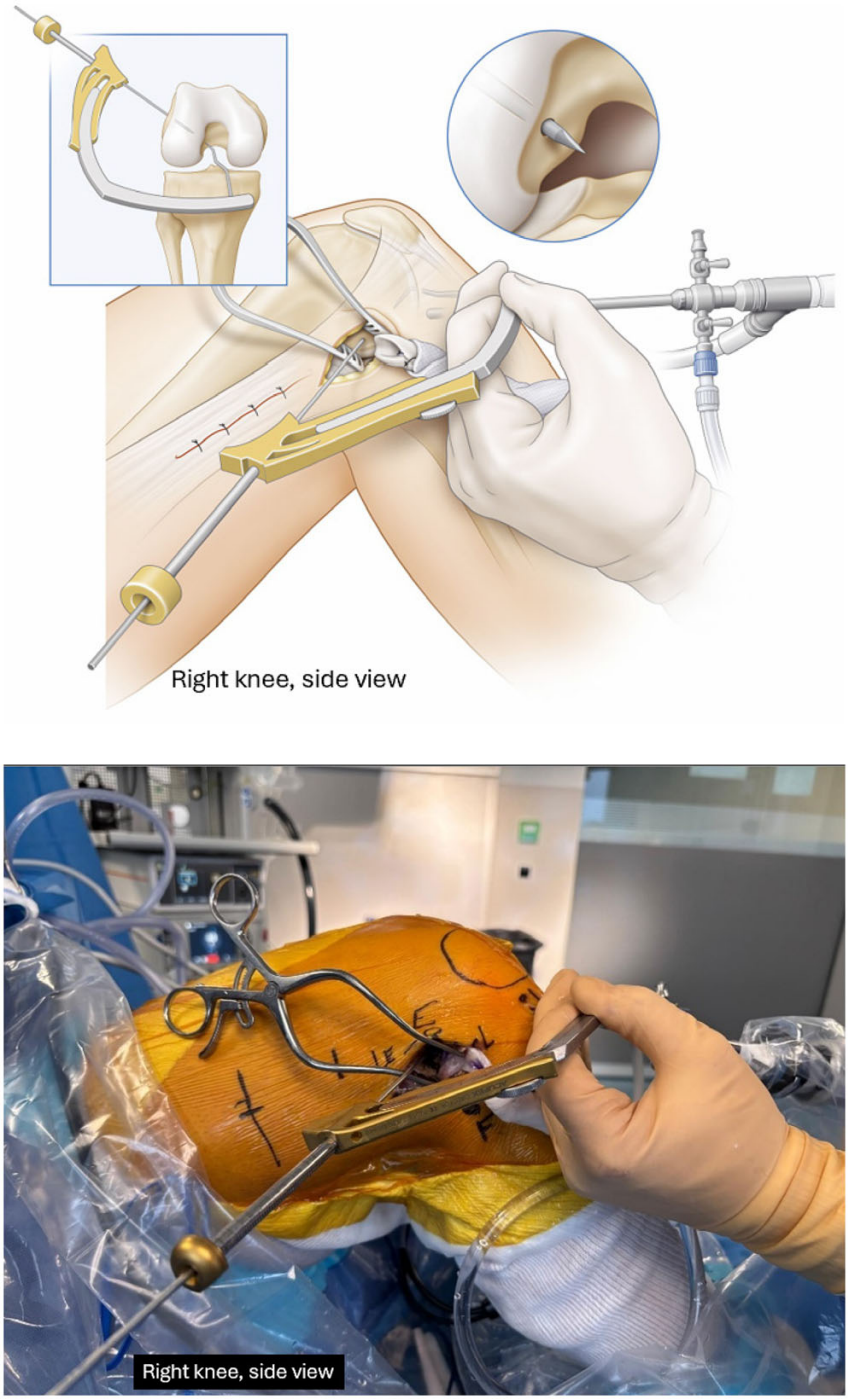
Right knee, supine position. Creating the femoral tunnel.

### Drilling the Tibial Tunnel

The tibial tunnel is created with a specific drill guide at 55°, a guide pin, and a drill. The tip of the tibial drill guide is inserted through the anteromedial portal and positioned at the ACL footprint. The tibial tunnel is first drilled using a 5‐mm drill bit, then drilled with a drill bit matching the measured diameter of the tibial end of the graft.

### Graft Placement

The graft is routed above the lateral collateral ligament to prevent increased loading of the lateral femorotibial compartment. The traction sutures of the graft are passed outside‐in through the femoral tunnel with a clamp and recovered intra‐articularly under arthroscopic control through the femoral and then tibial tunnels with arthroscopic grasping forceps (Figure 7). They are then pulled forward by cycling the knee. The graft is attached to the tibial tunnel with an absorbable interference screw (Biosure, London, United Kingdom), after tapping. The FL is closed by simple interrupted suturing using absorbable braided sutures (Ethicon) mounted on a FIRSTPASS MINI straight suture passer (Smith & Nephew, London, United Kingdom), allowing access to the edges of the fascia at a distance from the portal; the FL sutures are self‐blocking slip knots. The subcutaneous and cutaneous planes are then sutured (Figure 8). The strengths and limitations of the XO FAST technique are summarized in Table 1.

**FIGURE 7:**
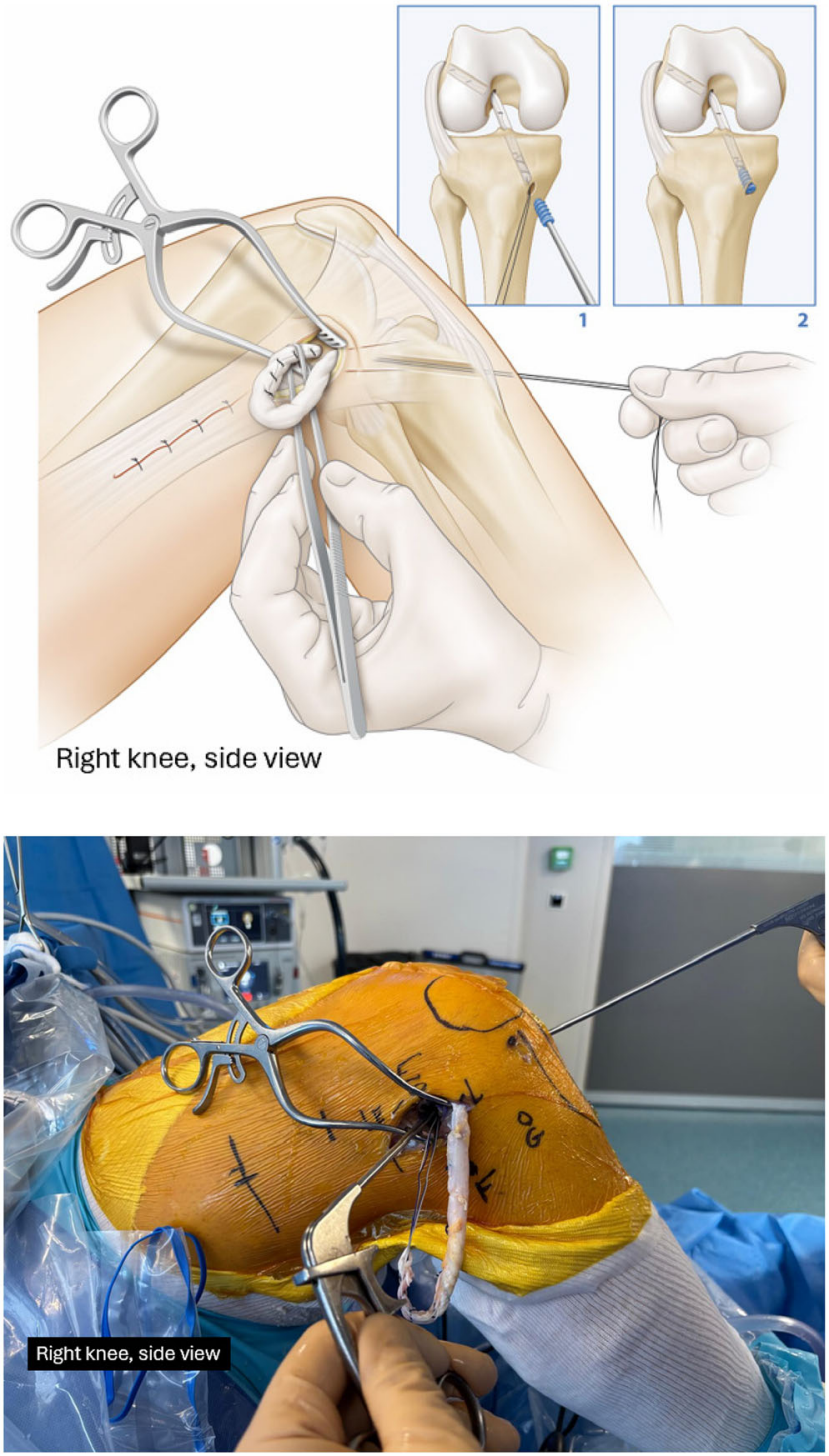
Right knee, supine position. Graft placement.

**FIGURE 8:**
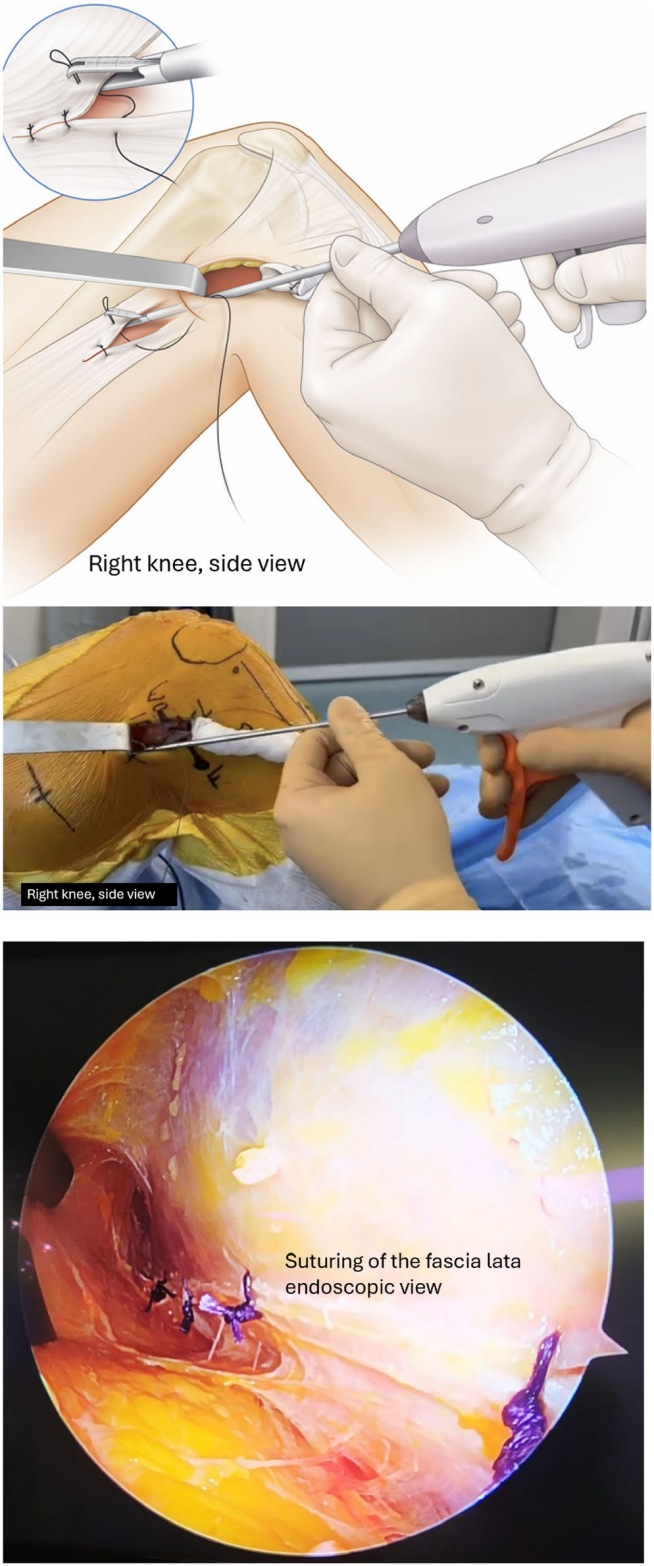
Right knee, supine position. Closing the fascia lata.

**TABLE 1:** Pearls and Pitfalls of the XO FAST Technique

**Pearls**	**Pitfalls**
Precise identification of the lateral epicondyle, fibular head, and Gerdy's tubercle improves reproducibility of the skin incision and graft harvesting	Inaccurate landmark identification may lead to nonisometric graft positioning and persistent rotational laxity
Respecting the described geometric measurements helps optimize tunnel positioning and graft isometry	Excessive anterior femoral tunnel placement may result in residual graft instability
Careful harvesting of the fascia lata band preserves graft integrity while minimizing donor‐site morbidity	Excessive thinning or aggressive harvesting of the fascia lata may weaken the graft
Use of a Smillie‐Trillat separator facilitates safe graft passage beneath the lateral structures	Inadequate soft tissue dissection may complicate graft passage
Verification of the isometric point before fixation helps maintain physiologic knee kinematics throughout flexion‐extension	Failure to verify isometry may produce abnormal graft tensioning during knee motion and persistent rotational laxity
Progressive and controlled femoral tunnel drilling decreases the risk of cortical breach	Aggressive reaming or incorrect drilling orientation may damage the lateral femoral condyle cortex
Using a single hamstring tendon helps minimize donor‐site morbidity	Inadequate graft diameter or poor graft preparation may compromise fixation strength
Combining ACL reconstruction with simultaneous extra‐articular lateral tenodesis improves rotational stability	Excessive graft constraint may limit internal tibial rotation and alter knee biomechanics
Maintaining appropriate graft tension during fixation optimizes rotational control while avoiding overconstraint	Excessive graft tension may restrict knee motion and increase lateral compartment pressure
Meticulous closure of the fascia lata reduces postoperative pain and donor‐site complications	Inadequate fascia lata closure may favor postoperative herniation or local discomfort

ACL, anterior cruciate ligament.

## DISCUSSION

This surgical technique reduces morbidity at the donor site during ACL reconstruction using a FL graft reinforced with the G (Table 2). The incision site for this minimally invasive surgical procedure is similar to that used for lateral tenodesis with the FL^10^ (Table 3).

**TABLE 2:** Advantages and Disadvantages of the Technique

**Advantages to the Technique**	**Disadvantages to the Technique**
Small incision site	Postoperative inflammation
Pediatric indication (preserves the epichondrial plate in the femur)	The length of surgery
Intra‐ and extra‐articular graft	Reinforcement by the gracilis/TQ/allograft
Preserves the semitendinosus	Learning curve
Revision ACL reconstructions	
Economical/low carbon footprint (1 single screw/graft)	
Reduces thigh muscle deconditioning	

ACL, anterior cruciate ligament; TQ, quadricipital tendon.

**TABLE 3:** XO FAST Versus Other Combined Intra‐ and Extra‐articular ACL Reconstruction Techniques

**Comparison Criteria**	**XO FAST**	**BTB + Lateral Tenodesis of the FL**	**STGT + Lateral Tenodesis of the FL**	**STGT Pediculated + Lateral Tenodesis of the FL**	**GT4/GT3 ALL**
Indication	Primary or Revision PEDIATRIC	Primary or revision	Primary, revision	Primary or revision	Primary, revision
Incision	Femur 3 cm/tibia 2 cm	Femur 2 cm/tibia 6 cm	Femur 2 cm/tibia 2 cm	Femur 2 cm/tibia 2 cm	Femur 2 cm/tibia 2 cm
	2 incisions	2 incisions	2 incisions	2 incisions	2 or 3 incisions
Harvesting	FL/gracilis	BTB/ITB	ST/gracilis/ITB	ST/gracilis/ITB	ST/gracilis
Fixation	Pedicled to the femur	Free graft	Free graft	Pedicled to the tibia	Free graft
Femoral tunnel	Outside‐In	Outside‐in or Inside‐out	Outside‐in or Inside‐out	Outside‐in or Inside‐out	Outside/in or Inside/out
Graft	1	3	3	3	4
Carbon footprint	+	++	++	++	++++
Cost	+	++	++	++	++++

ACL, anterior cruciate ligament; BTB, bone to bone; FL, fascia lata; ITB, iliotibial band; ST, semi tendinosus tendon; STGT, semi tendinosus gracilis tendon.

ACL reconstruction using an FL graft reinforced with the G keeps the extensor apparatus intact and preserves the semitendinosus tendon, favoring muscle and hamstring recovery. This type of reconstruction reduces comorbidity from harvesting with postoperative quadriceps and hamstring recovery and has very good isokinetic test results: the slow‐speed quadriceps deficit was 22.5% at 6 months and 11% at 1 year after surgery; the slow‐speed hamstring deficit was 7% at 6 months and 6.4 % at 1‐year follow‐up.^11^

Another important advantage of this technique is to create an extra‐articular graft at the same time as the ACL reconstruction. This allows for better control of medial rotation^12^, ^13^ to decrease the risk of an ACL retear^9^ and to limit the risk of an unsuccessful meniscal suture.^14^

Finally, this technique is economical and ecological because both the ACL reconstruction and the extra‐articular graft can be performed using a single interference screw for the tibial tunnel with no femoral fixation. It is also the ACL reconstruction technique with the lowest carbon footprint.^15^

This technique with an extra‐articular graft without femoral fixation is indicated for primary pediatric surgery with open or closed growth plates. The absence of femoral fixation and the single femoral tunnel is also a technical advantage for revision surgery.

In conclusion, this minimally invasive ACL reconstruction technique associating an FL graft augmented with the G is an improvement to the classic procedure. This technique limits incision site morbidity during harvesting of the FL and has all the other advantages of FL ligament reconstruction, including rotational stability and preservation of the extensor apparatus and the semitendinosus. It is indicated in cases of open or closed physes for primary or revision procedures.

## DISCLOSURES

The author (A.H.) declares the following financial interests/personal relationships which may be considered as potential competing interests: A.H. receives consulting fees from Arthrex. The other authors (P.D‐A., S.H., Y.B.) declare that they have no known competing financial interests or personal relationships that could have appeared to influence the work reported in this article.
